# Service and treatment engagement of people with very late-onset schizophrenia-like psychosis

**DOI:** 10.1192/pb.bp.115.051599

**Published:** 2016-08

**Authors:** Chun Chiang Sin Fai Lam, Suzanne J. Reeves, Robert Stewart, Robert Howard

**Affiliations:** 1 South London and Maudsley NHS Foundation Trust; 2 King's College London; 3 University College London

## Abstract

**Aims and method** Electronic patient records were used to investigate the level of engagement and treatment that patients with very late-onset schizophrenia-like psychosis (VLOSLP) had with mental health services.

**Results** Of 131 patients assessed and diagnosed, 63 (48%) were taking antipsychotic treatment at 3 months, 46 (35%) at 6 months and 36 (27%) at 12 months. At discharge from mental health services, 54% of patients had failed to engage with services or became lost to follow-up, 18% had engaged with services but were not taking antipsychotic medication and only 28% were taking treatment.

**Clinical implications** Results showed that less than half of the patients with VLOSLP were commenced on antipsychotic treatment and less than a third remained on treatment at 1 year or at point of discharge. This highlights the need for services to consider being more assertive in taking potentially effective treatment to this patient group.

A Cochrane review^1^ has concluded that there is no good randomised clinical trial evidence on which to base treatment guidelines for patients with very late-onset schizophrenia-like psychosis (VLOSLP),^2^ yet open treatment with atypical antipsychotics has been associated with improvements in symptoms at least as good as those seen in younger patients with schizophrenia or patients with early-onset schizophrenia who have grown old,^3–5^ and antipsychotic treatment is the cornerstone of care pathways. Little is known about how many patients seen within specialist mental health services are engaged by those services and given antipsychotic treatment. To address this, we conducted a retrospective electronic patient record search.

## Method

### Participants

We used the Clinical Record Interactive Search (CRIS) system, developed within the National Institute for Health Research (NIHR) Mental Health Biomedical Research Centre, South London and Maudsley NHS Foundation Trust, to extract the records of all patients aged 60 years and above with a diagnosis of schizophrenia, schizotypal and/or delusional disorders (ICD-10 F20–F29)^6^ in contact with services between 1 January 2007 and 21 August 2014. All identified patients' notes were reviewed and those who scored 24 or less on the standardised mini-mental state examination^7^ or had a concurrent diagnosis of an organic mental disorder (ICD-10 F00–F09)^6^ were excluded. Other exclusion criteria included any evidence that psychosis onset had been before the age of 60, insufficient recorded information to confirm diagnosis and less than 1 year of follow-up completed at time of CRIS search.

### Events

The first face-to-face contact of mental health services with a patient was regarded as the start point of each service episode. Records were reviewed at 3 months to assess initial engagement with services. If a patient was discharged before 3 months, the last documented episode was used as an alternative. Two further engagement points, at 6 months and 12 months, were also examined. The length of each episode was also extracted and the level of engagement at the point of discharge from services was assessed.

Ethics approval for CRIS was given by the National Research Ethics Service Oxford REC C.

## Results

**Fig. 1:**
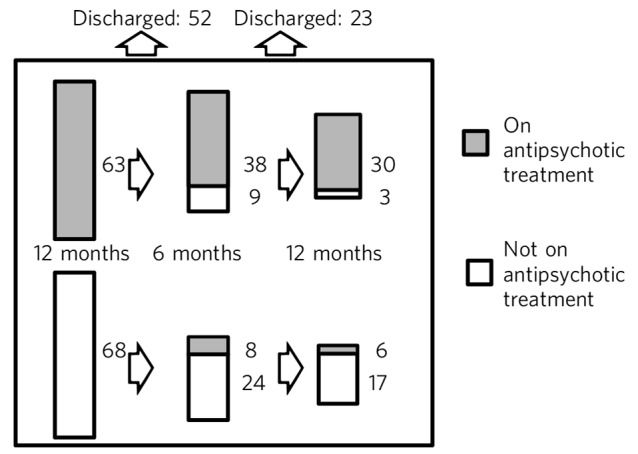
Service and antipsychotic treatment engagement over 12 months.

Search of the CRIS system initially identified 635 patients. Following inspection of the individual records, patients were excluded because of incorrect coding (*n* = 45), cognitive impairment (*n* = 216), onset of psychosis before age 60 years (*n* = 150), insufficient information to confirm diagnosis (*n* = 32) or in contact with services for less than 12 months preceding the search date (*n* = 61). Of the remaining 131 patients, 84 (64.1%) were female, 40 (30.5%) were White British, and 35 (26.7%) were living with a partner or family member. The extent of engagement with services and antipsychotic treatment at 3, 6 and 12 months is shown in Fig. 1. At the point of discharge from specialist services back to primary care, 65 patients (54%) had been lost to follow-up or had not engaged with specialist services, 22 (18%) had engaged with services but were not receiving antipsychotic treatment, and 33 (28%) were taking antipsychotic treatment. The rest were still receiving active specialist service follow-up. Twenty-six (19.8%) patients were treated compulsorily under a section of the Mental Health Act 1983, and neither male gender (odds ratio (OR) 1.15: 95% CI 0.47–2.80) nor membership of a Black or minority ethnic group (OR = 1.25: 95% CI 0.52–3.02) significantly influenced Mental Health Act use.

## Discussion

The diagnostic concept of VLOSLP emerged from an international consensus meeting held to advance research on a patient group with first onset of delusions and/or hallucinations after the age of 60 years, in the absence of affective disorder or demonstrable brain disease such as dementia.^2^ The illness is viewed as a functional psychosis with symptoms that will respond to antipsychotic drugs.^2–5^ Our data show that less than half of patients with VLOSLP were commenced on antipsychotic treatment and less than a third remained on treatment at 1 year or at the point of discharge from services. This is a surprising, even disappointing, result. An important barrier to acceptance of antipsychotic treatment by patients with VLOSLP is the low level of insight into presence of mental health difficulties or need for treatment.^8^ Although treatment-related decision-making capacity has not been specifically investigated in VLOSLP, studies of middle-aged and older patients with schizophrenia have suggested that cognitive test scores, rather than psychopathology ratings, associate most strongly with the understanding and reasoning components of capacity.^9^ Patients with VLOSLP do not, however, have demonstrably abnormal brain imaging^10^ and do not inevitably progress to develop dementia,^11,12^ so cognitive impairment is unlikely to explain their inability to evaluate their illness or the need for treatment. Reluctance by clinicians to use mental health law to deliver compulsory treatment when patients will not accept the case for antipsychotic treatment is indicated by the small percentage of cases where this happened. Although there are limited studies to compare this with, it is in keeping with the lower rates of patients on longer-term detention within the above-65 population.^13^ This may reflect a view that elderly patients with psychosis are somehow less ‘risky’ and that a failure to enforce treatment is therefore justifiable. In fact, untreated symptoms of VLOSLP are frightening and disabling, lead patients to place themselves at significant risk and damage relationships with family and neighbours. Comparably low levels of psychosis treatment in younger people would be completely unacceptable and specialist mental health services for older people should actively consider whether they should be more assertive in taking potentially effective treatment to this vulnerable patient group.
